# Conditional and inducible transgene expression in endothelial and hematopoietic cells using Cre/*loxP* and tetracycline-off systems

**DOI:** 10.3892/etm.2014.1965

**Published:** 2014-09-15

**Authors:** JU LIU, URBAN DEUTSCH, IRIS FUNG, CORRINNE G. LOBE

**Affiliations:** 1 Laboratory of Microvascular Medicine, Medical Research Center, Shandong Provincial Qianfoshan Hospital, Shandong University, Jinan, Shandong 250014, P.R. China; 2 Molecular and Cellular Biology Division, Sunnybrook Health Science Centre, Toronto, Ontario M4N 3M5, Canada; 3 Department of Medical Biophysics, University of Toronto, Toronto, Ontario M5G 1L7, Canada; 4 Theodor-Kocher-Institute, University of Berne, Berne CH-3012, Switzerland

**Keywords:** Cre/*loxP* system, tetracycline, endothelial cells, hematopoietic cell, transgenic mice

## Abstract

In the present study, the tetracycline-off and Cre/*loxP* systems were combined to gain temporal and spatial control of transgene expression. Mice were generated that carried three transgenes: Tie2-tTA, tet-O-Cre and either the ZEG or ZAP reporter. Tie2-tTA directs expression of tetracycline-controlled transactivator (tTA) in endothelial and hematopoietic cells under the control of the Tie2 promoter. Tet-O-Cre produces Cre recombinase from a minimal promoter containing the tet-operator (tetO). ZEG or ZAP contains a strong promoter and a *loxP*-flanked stop sequence, followed by an enhanced green fluorescence protein (EGFP) or human placental alkaline phosphatase (hPLAP) reporter. In the presence of tetracycline, the tTA transactivator produced by Tie-2-tTA is disabled and Cre is not expressed. In the absence of tetracycline, the tTA binds tet-O-Cre to drive the expression of Cre, which recombines the *loxP* sites of the ZEG or ZAP transgene and results in reporter gene expression. In the present study, the expression of the ZEG or ZAP reporter genes in embryos and adult animals with and without tetracycline treatment was examined. In the presence of tetracycline, no reporter gene expression was observed. When tetracycline was withdrawn, Cre excision was activated and the reporter genes were detected in endothelial and hematopoietic cells. These results demonstrate that this system may be used to bypass embryonic lethality and access adult phenotypes.

## Introduction

The circulatory system is the first functional system formed during mammalian embryogenesis, beginning with the aggregation of hemangioblasts, the common precursors of endothelial and hematopoietic cells, shortly following gastrulation. The hemangioblasts differentiate into several distinct cell lineages that form the network of blood vessels, the heart, circulating blood cells and supporting tissues. A number of molecules and signal transduction pathways are temporally and quantitatively regulated to maintain normal cardiovascular development (1,2). These include vascular endothelial growth factor (VEGF), basic fibroblast growth factor (bFGF), the angiopoietins/Tie pathway, the ephrins/Eph pathway and the DSL/Notch pathway. All of these pathways have been studied *in vivo* using traditional knockout, knock-in and transgenic methods. In the majority of cases, these gene mutations induce embryonic lethality between E9.5 to E11.5 (3–5). However, the embryonic lethal phenotypes are similar and suggest that disruption to any of these pathways results in defects in the remodeling and maturation of the vasculature (6,7). The embryonic lethal phenotypes hinder the investigation of the function of these genes in later development, including possible roles in the determination of vessel identity, hematopoiesis, endothelial-mesenchymal transition and pericyte recruitment.

A number of techniques have been developed to achieve cell type-/tissue-specific transgene expression. The first established inducible system was the *lac* repressor system regulated by isopropyl-β-D-thiogalactopyranoside (IPTG) (8,9). Currently, the Cre/*loxP* and tetracycline systems are the most widely used systems to control gene expression. Cre recombinase is a bacteriophage enzyme that recognizes two 34-base-pair *loxP* sites and excises the DNA between them (10,11). Conditional gene deletion and expression strategies have been developed by placing *loxP* sites around an essential part of a gene of interest or separating an ectopic promoter from the coding region of a gene by *loxP*-flanked stop sequences (12–14). In these configurations, the gene alteration is silent prior to the excision of the *loxP* sites. Following the introduction of Cre recombinase under a tissue-specific promoter, the *loxP*-flanked sequence is excised and gene inactivation or transgene expression occurs specifically in the tissues where Cre is expressed. While the Cre/*loxP* system provides spatial control of gene alteration, the tetracycline-inducible system provides temporal regulation. Under this system, a fusion protein composed of the VP16 transactivator and either the tetracycline repressor (tTA) or reversed tetracycline repressor (rtTA) can be inhibited (tet-off) or activated (tet-on) by tetracycline, respectively (15,16). Therefore, the addition or withdrawal of tetracycline controls the activity of the transactivator, which then regulates the expression of a given gene from a promoter containing the tet-operator (tetO) sequences.

In the present study, a strategy was developed in which the Cre/*loxP* and tet-off systems were combined to allow for spatial and temporal regulation of transgene expression *in vivo*. In this system, three transgenes are utilized. The first transgene drives the expression of a tetracycline-controlled transactivator (tTA) under the control of the Tie2 promoter, which allows selective transgene expression only in endothelial and hematopoietic cells. The second transgene expresses the Cre recombinase gene from a minimal promoter containing tet-operator (tetO) sequences (17). The third transgene is the ZEG or ZAP reporter, which has a strong global promoter separated from an enhanced green fluorescence protein (EGFP) or human placental alkaline phosphatase (hPLAP) reporter gene by a *loxP*-flanked stop sequence. The stop sequence contains a *lacZ*-neomycin resistance fusion (β-geo) gene with translation and transcription stops (13,18). With the combination of these transgenes, Cre expression does not occur in the presence of tetracycline and consequently the reporter genes are not expressed. At any point during embryonic development or adulthood, Cre expression can be activated through the removal of tetracycline, leading to Cre excision and expression of the reporter genes.

The advantage of combining the tetracycline-inducible and Cre/loxP systems is that once Cre is activated, it will excise the target transgene and make a permanent genetic alteration. The reporter gene will be expressed in the Cre-expressing cells and in all the progeny of that cell, even if the inducing condition is removed. Thus a limited number of cells can be marked for reporter gene expression for applications such as mosaic analysis. This also represents a valuable approach to the modelling of cancer, where a limited number of cells in an organ undergo the critical oncogenic mutations and those cells then go on to form a tumor. In developing cancer treatments and testing them, this type of model may be useful to ascertain the effectiveness of a treatment at eliminating the cancerous cells. By contrast, in systems where only an inducing agent, for example tetracycline or tamoxifen, is used, expression of the target transgene requires the continuous application of the inducing condition, and that continuous application leads to an endless supply of oncogene expressing cells; thus, it is difficult to determine whether a treatment eliminates the oncogenic cells.

## Materials and methods

### Transgenic mice

The Tie2-tTA mice (provided by Dr Andras Nagy; Lunenfeld-Tanenbaum Research Institute, Toronto, Canada) express the tTA in endothelial and hematopoietic cells under the control of a 2.1 kb Tie2 promoter fragment and a 10 kb fragment carrying an enhancer present in the first intron of Tie-2 (19). The tet-O-Cre mice (also provided by Dr Andras Nagy), carry the Cre coding sequence downstream of a minimal cytomegalovirus (CMV) promoter and tetracycline operator. ZEG and ZAP reporter mice were constructed in our laboratory, as previously described (13,18). The mice have a strong CMV-enhancer/chicken β-actin promoter followed by a *loxP*-flanked stop sequence consisting of a *lacZ*-neomycin resistance fusion (β-geo™) gene and three polyadenylation sequences. The *loxP*-flanked stop is followed by hPLAP in ZAP mice and EGFP in ZEG mice. The Animal Care Committee of Sunnybrook Research Institute (Toronto, Canada) approved all animal experiments carried out in the present study.

### Mouse genotyping

The ZEG and ZAP mice were genotyped by *lacZ* staining of ear biopsies as previously described (13,18). Tie2-tTA and tet-O-Cre mice were genotyped using PCR using genomic DNA isolated from mouse ear biopsies. Genomic DNA was extracted from the biopsies using a REDExtract-N-Amp^™^ Tissue PCR kit (Sigma-Aldrich, St. Louis, MO, USA) following the manufacturer’s instructions. For each sample analyzed, ~100 ng of DNA was amplified using a S1000^™^ Thermal Cycler (Bio-Rad, Hercules, CA, USA). The primers used were as follows: Cre forward, 5′-AATTTACTGACC GTACACCA-3′; Cre reverse, 5′-CGCCGCATAACCAGT GAAAC-3′; tTA forward, 5′-CTCACTTTTGCCCTTTAG AA-3′; tTA reverse, 5′-GCTGTACGCGGACCCACTTT-3′. The thermal-cycle program was as follows: 94°C for 5 min (1 cycle), 94°C for 30 min, 55°C for 1 min, 72°C for 1 min (30 cycles), 72°C for 5 min (1 cycle).

### Alkaline phosphatase (AP) staining

AP staining was performed as previously described (14). Briefly, pregnant females were euthanized by cervical dislocation and the uterus was removed and washed in cold phosphate-buffered saline (PBS). Embryos were dissected out of the uterus and parts of the yolk sacs were removed for genotyping, whereas the embryos were fixed in *lacZ* fix solution (0.2% glutaraldehyde, 50 mM EGTA, pH 7.3, 100 mM MgCl_2_ in 100 mM sodium phosphate, 0.02% NP-40 and 0.01% sodium deoxycholate, pH 7.3) for 15 min. For the cross sections, the slides were prepared as described below. Embryos or slides were then washed in PBS and endogenous APs were inactivated by incubation in PBS at 70–75°C for 30 min. Following washing in AP buffer (100 mM Tris-HCl, pH 9.5, 100 mM NaCl, 10 mM MgCl_2_) for 10 min, the samples were stained with AP staining solution [(100 mM Tris-HCl, pH 9.5, 100 mM NaCl, 50 mM MgCl_2_, 0.01% sodium deoxycholate, 0.02% NP-40, 337 mg/ml nitroblue tetrazolium salt (Roche Diagnostics, Basel, Switzerland), and 175 mg/ml 5-bromo-4-chloro-3-indolyl phosphate, toluidinium salt (Roche Diagnostics)]. The staining reaction was allowed to proceed for 10–30 min at room temperature. The samples were then washed in PBS and stored at 4°C.

### Immunohistochemistry

The tissue samples were dissected in cold PBS and fixed in 4% paraformaldehyde overnight, washed in PBS three times for 15 min and cryoprotected in 15% sucrose in PBS for 1 h at 4°C followed by 30% sucrose in PBS overnight at 4°C. The samples were then incubated in Tissue-Tek OCT (Sakura Finetek USA Inc, Torrance, CA, USA) at 4°C for 4 h prior to embedding in OCT over dry ice. The frozen blocks were cryosectioned at 7 μm, placed onto L-polylysine-coated slides (Thermo Fisher Scientific, Waltham, MA, USA), and dried for 1–4 h at room temperature prior to storage at −20°C. For immunostaining, the sections were quenched sequentially in 3% hydrogen peroxide and blocked with diluted 10% normal goat serum (for EGFP) or 5% normal rabbit serum [for platelet-endothelial cell adhesion molecule (PECAM)]. Slides were then incubated at 4°C overnight with the primary antibodies, which comprised anti-PECAM-1 monoclonal antibody (1:100; BD Pharmingen, San Diego, CA, USA) and anti-GFP monoclonal (3E6) antibody (1:2,000; Molecular Probes, Eugene, OR, USA). The next day, the slides were exposed for 30 min to biotinylated secondary antibodies including rabbit anti-rat or goat anti-rabbit (1:200; Vector Laboratories, Inc., Burlingame, CA, USA). The peroxidase activities were visualized using streptavidin-horseradish peroxidase together with the diaminobenzidine detection system (Vector Laboratories, Inc.). Finally, slides were washed and counterstained with hematoxylin (Surgipath; Leica Microsystems, Wetzlar, Germany). Images of slides were captured by photography using a Leica DFC300 camera with Leica FireCam 120 program.

### Flow cytometry

Single cell suspensions were prepared by dissociating tissues in fluorescence-activated cell sorting (FACS) buffer (PBS with Ca^2+^/Mg^2+^, 0.1% NaN_3_ and 5% FCS) and filtering through a 40-μm cell filter. Spleen samples were treated with freshly prepared red blood cell lysis buffer (9:1 mix of 0.83% NH_4_Cl and 1 m Tris-HCl pH 7.65) and refiltered. Samples were resuspended in FACS buffer supplemented with 1 mg/ml of propidium iodide. Appropriate isotype controls were included with each experiment. Fluorescence of EGFP was detected on the FL-1 channel of a FACSCalibur instrument (BD Biosciences, Franklin Lakes, NJ, USA). Results were then analyzed using FlowJo (TreeStar Inc., San Carlos, CA, USA).

## Results

### Tie2-tTA/tet-O-Cre transgene combination activates Cre excision in endothelial and hematopoietic cells

**Figure 1:**
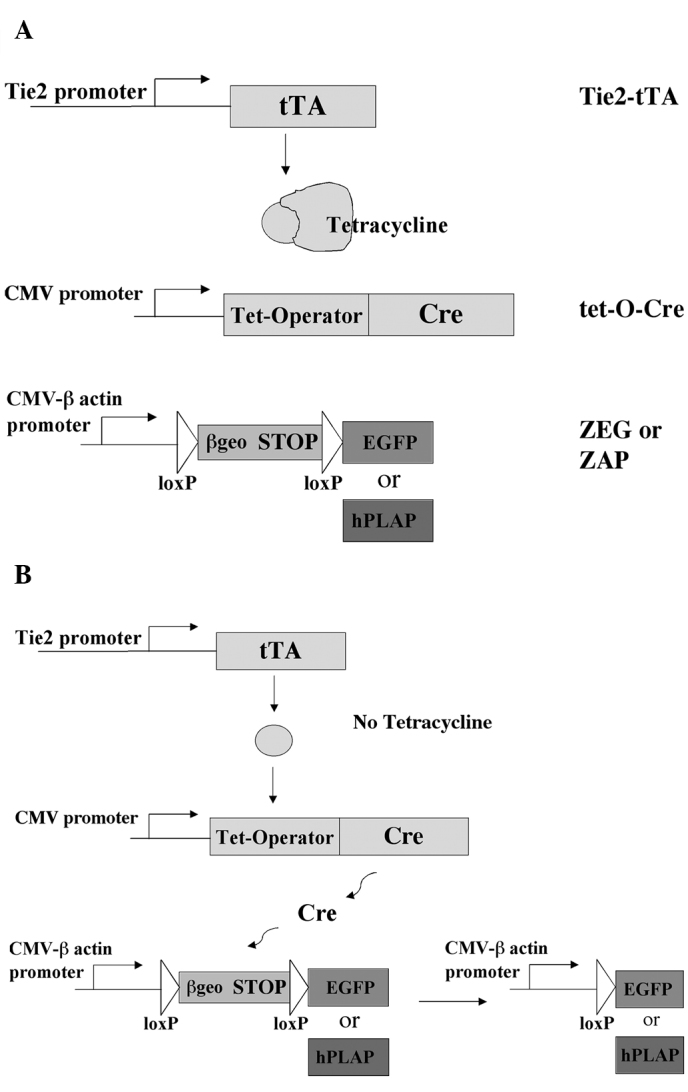
Tetracycline-Cre inducible system. (A) Tie2-tTA/ZEG or Tie2-tTA/ZAP mice were crossed with tet-O-Cre mice. In the presence of tetracycline, the triple transgenic embryos (Tie2-tTA/tet-O-Cre/ZEG) express tTA in endothelial cells and hematopoietic cells, but the tetracycline-bound tTA does not bind the tet operator. (B) When tetracycline is withdrawn, the tTA binds to the tet operator, which induces the activation of Cre expression and results in the deletion of the STOP sequence and expression of the reporter [enhanced green fluorescence protein (EGFP) or human placental alkaline phosphatase (hPLAP)].

**Figure 2:**
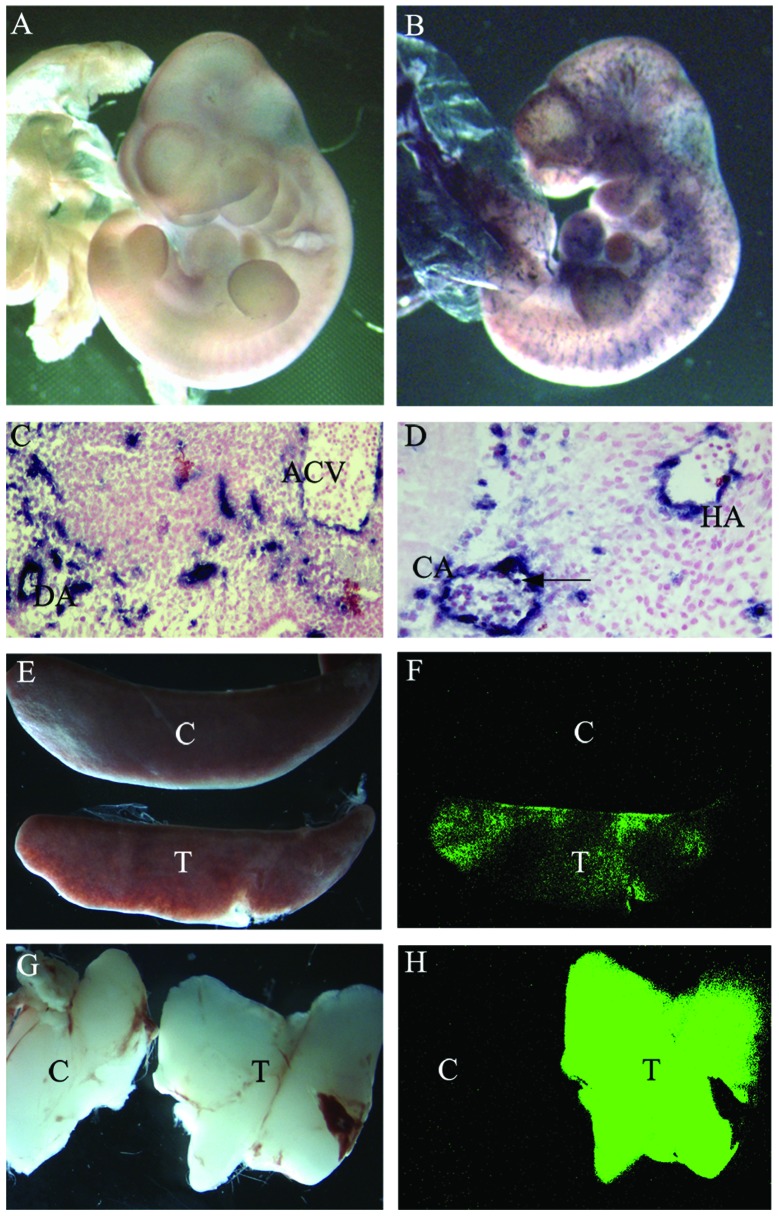
Measurement of Cre excision in the absence of tetracycline treatment. (A and B) Whole-mount AP staining of (A) E10.5 control embryos and (B) E10.5 Tie2-tTA/tet-O-Cre/ZAP embryos; (C and D) AP staining of sections of E11.5 Tie2-tTA/tet-O-Cre/ZAP embryos. Arrows indicate AP positive hematopoietic cells. (E–H) EGFP fluorescence. (E) Bright field view of spleens from control and Tie2-tTA/tet-O-Cre/ZEG mice and (F) the same spleens under GFP light. The spleen from a control mouse is dark, whilst the spleen from a triple transgenic mouse exhibits green fluorescence. (G) Bright field view of thymuses from control and Tie2-tTA/tet-O-Cre/ZEG mice and (H) the same spleens under GFP light. The thymus from a control mouse is dark, whilst the thymus from a Tie2-tTA/tet-O-Cre/ZEG mouse exhibitis green fluorescence. AP, alkaline phosphatase; DA, dorsal aorta; ACV, anterior cardinal vein; CA, cortical artery; HV, head vein; C, control mice; T, triple transgenic Tie2-tTA/tet-O-Cre/ZEG mice; EGFP, enhanced green fluorescence protein.

The efficiency of Cre excision activated by the Tie2-tTA/tet-O-Cre transgene combination was analyzed by maintaining mice in the absence of tetracycline (Fig. 1B). ZAP was initially used as the reporter mouse line (13,20). Tet-O-Cre male mice were bred with Tie2-tTA/ZAP female mice and embryos were collected at E8.5, 9.5, 10.5 and 11.5. The embryos and yolk sacs of the entire litter were stained for AP activity. Part of the yolk sac was also genotyped by PCR to identify triple transgenic embryos. At E8.5, no staining was observed in the Tie2-tTA/tet-O-Cre/ZAP embryos and yolk sac. At E9.5, a substantial number of cells positively stained for AP were observed in the yolk sac and the heart (data not shown). At E10.5, the majority of the yolk sac stained for AP (Fig. 2B) and AP positive vessels were also observed within the cranium and somites (Fig. 2B). At E11.5 the yolk sac strongly stained for AP, while the vessels in the embryos were already deep beneath the tissue at this stage and were not visible clearly.

The embryos were frozen and transversely sectioned to observe the AP staining within the embryo. Virtually all endothelial cells of Tie2-tTA/tet-O-Cre/ZAP embryos in sections showed positive AP staining at E11.5 (Fig. 2C and D). The endogenous Tie2 receptor is expressed not only in endothelial cells, but also in hematopoietic cells, which aggregate and adhere to the endothelial cells of arteries (21). It was observed that a fraction of hematopoietic cells were positively stained for AP on E11.5 within the arteries, whereas hematopoietic cells in the veins were not stained (Fig. 2C and D). Similar observations have been previously described for Tie2-Cre/CAG-CAT-Z embryos (22).

While AP expression of Tie2-tTA/tet-O-Cre/ZAP embryos was restricted at E8.5 and E9.5, Tek-tTA/Tet-O-*LacZ* embryos showed *lacZ* expression beginning at E8.5 (Tek is a Tie2 promoter) (23). This supports previous observations that Cre excision and resultant reporter gene expression may not occur until 1–2 days after Cre expression is initiated (20).

ZEG reporter mice were used to analyze the expression within adult tissues since the second reporter EGFP may be visualized in live animals and cells either directly or using flow cytometry (18,20). Tie2-tTA/ZEG mice were bred with tet-O-Cre mice and triple transgenic Tie2-tTA/tet-O-Cre/ZEG pups were obtained, which displayed light green fluorescence at birth, particularly in the ears and the tail (data not shown). These pups were later confirmed to be triple transgenic using PCR and *lacZ* staining of ear biopsies (*lacZ* is expressed from the non-excised allele of the ZEG transgene). Although tTA has been reported to be toxic in mammalian cells (15), the triple transgenic embryos did not exhibit any obvious phenotype. All of the triple transgenic mice showed GFP expression at birth and in hematopoietic organs. Hematopoietic tissues, including the thymus and spleen, were also GFP fluorescent (Fig. 2F and H). In frozen sections of highly vascularized tissues, including the lung and kidney, blood vessel endothelial cells also exhibited green fluorescence (data not shown). These results confirm that the Tie2-tTA/tet-O-Cre transgenes mediate Cre excision in endothelial and hematopoietic cells.

### Cre excision is delayed by maintenance on doxycycline and activated by doxycycline withdrawal

Once it was established that the triple transgenic system produced reporter gene expression in endothelial and hematopoietic cells in the absence of tetracycline, it was then determined whether reporter gene expression would be inhibited in the presence of tetracycline. It was hypothesized that tetracycline would inhibit tTA activation of the tet-O-Cre transgene; thus, Cre would not be expressed, and consequently the AP and EGFP reporter genes would not be expressed (Fig. 1A).

Breeding pairs of tet-O-Cre males and Tie2-tTA/ZEG females were maintained on 0.1 mg/ml doxycycline supplied in the drinking water from the time they were mated. At this level, doxycycline, a tetracycline analogue, is sub-therapeutic but sufficient to repress tTA (23). Following doxycycline treatment, breeding pairs did not produce any pups exhibiting green fluorescence, although Tie2-tTA/tet-O-Cre/ZEG triple transgenic pups (identified by PCR genotyping) were observed in a normal Mendelian ratio. The thymus, spleen, kidney and liver of these newborn triple transgenic pups did not show any GFP fluorescence, indicating that tTA was repressed by doxycycline.

**Figure 3:**
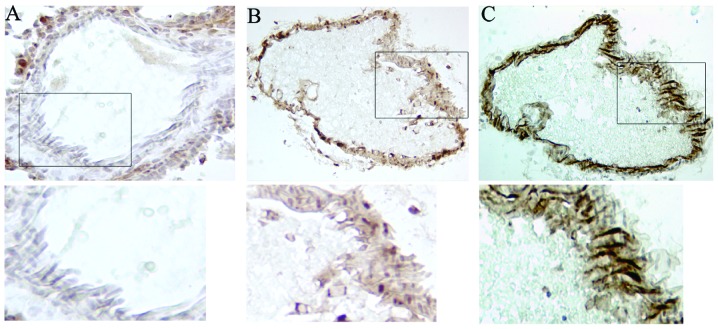
Measurement of Cre Excision in endothelial cells following tetracycline withdrawal. Breeding pairs were maintained on doxycycline, a tetracycline analogue, during pregnancy. Doxycycline was withdrawn the day after the litters were born and tissues were analyzed after 28 days. (A and B) EGFP immunostaining of blood vessels from (A) a control mouse and (B) a Tie2-tTA/tet-O-Cre/ZEG mouse, which showed mosaic EGFP staining in endothelial cells. (C) Platelet-endothelial cell adhesion molecule staining of a serial section of (B). EGFP, enhanced green fluorescence protein.Upper panel magnification, ×100; lower panel magnification, X400.

**Figure 4:**
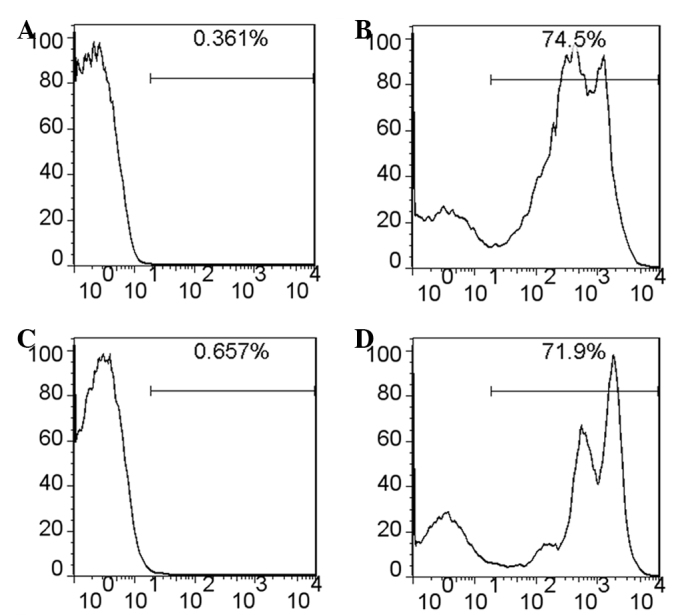
Measurement of Cre excision in hematopoietic cells following tetracycline withdrawal. (A–D) Fluorescence-activated cell sorting analysis to measure enhanced green fluorescence protein expression in (A) the thymus from a control mouse; (B) the thymus from a Tie2-tTA/tet-O-Cre/ZEG mouse; (C) the spleen from a control mouse and (D) the spleen from a Tie2-tTA/tet-O-Cre/ZEG mouse.

In some litters, doxycycline was withdrawn two days after the pups were born and pups were sacrificed after 28 days in order to determine whether Cre excision and subsequent EGFP expression could be obtained by postnatal doxycycline withdrawal (Fig. 1B). Mosaic expression of EGFP was observed in endothelial cells in various organs, as demonstrated by comparing immunostaining for EGFP and the endothelial marker PECAM-1 staining in serial sections of liver (Fig. 3B and C). With removal of doxycycline, Tie2-tTA/tet-O-Cre/ZEG triple transgenic thymus showed green fluorescence and EGFP^+^ cells were detected in the spleen and thymus using flow cytometry (Fig. 4B and D). Therefore, removal of doxycycline efficiently led to Cre expression and excision and consequent expression of the EGFP reporter.

## Discussion

Recently, a number of studies have attempted to characterize the genes involved in pathological processes of adult vasculature (24–26). The Cre conditional system combined with the inducible tTA system used in the present study enables the deletion of a gene or expression of a mutated gene at any time point during the lifespan of the mice. In particular, it allows studies of gene functions at stages of development after which deletion or misexpression is normally lethal. For example, deletion of the Notch1 receptor has been found to be embryonic lethal at E9.5, thus limiting the understanding of its role during early embryogenesis (3,6). Using this system, this problem may be overcome, and the Notch1 receptor can be deleted after E10.5 to study its potential function in adult angiogenesis, which has not been possible to investigate directly by gene targeting studies.

Unlike endothelial cells, which remain stable in the majority of adult organs, the hematopoietic system is a developing system (27,28). Mutation of a specific gene at different ages can result in different types of diseases or prognosis (29–31). By controlling the temporal activation of transgene expression, this inducible system may be useful in order to investigate gene function in hematopoiesis, leukemogenesis and lymphomagenesis, in particular disease-associated genes where genetic modification results in embryonic lethality.

In the present study, mosaic reporter gene expression occurred in endothelial and hematopoietic tissues in adult triple transgenic mice following doxycycline withdrawal. This may result from incomplete Cre activity, transgene silencing and/or reduced activation of the Tie2 promoter in adults. Mosaic expression has been observed in the majority of tetracycline-regulated models and also in other inducible systems (20,32). However, gene functional studies can be conducted by comparison with appropriate controls and quantitative studies may be achieved through a variety of techniques.

In conclusion, it was demonstrated in the present study that a tetracycline-regulated Cre/*loxP* system may be used to conditionally express transgenes in endothelial and hematopoietic cells. The combination of the Cre/*loxP* and tetracycline-inducible systems to activate transgene expression can be used in other tissues by incorporating different promoters to drive the expression of tTA or rtTA (17,32). The application of this system may improve our ability to bypass embryonic lethal phenotypes and study gene function in adult mice. Furthermore, it may provide valuable animal models for therapeutic development.
